# Rheumatoid Arthritis and Cardiovascular Risk: Retrospective Matched-Cohort Analysis Based on the RECORD Study of the Italian Society for Rheumatology

**DOI:** 10.3389/fmed.2021.745601

**Published:** 2021-10-05

**Authors:** Lisa Argnani, Anna Zanetti, Greta Carrara, Ettore Silvagni, Giulio Guerrini, Antonella Zambon, Carlo Alberto Scirè

**Affiliations:** ^1^Department of Experimental, Diagnostic and Specialty Medicine, University of Bologna, Bologna, Italy; ^2^Epidemiology Unit, Italian Society for Rheumatology, Milan, Italy; ^3^Department of Statistics and Quantitative Methods, Division of Biostatistics, Epidemiology and Public Health, University of Milano-Bicocca, Milan, Italy; ^4^Rheumatology Unit, Department of Medical Sciences, University of Ferrara and Azienda Ospedaliero-Universitaria S.Anna, Cona, Italy; ^5^Biomedical and Biotechnological Science at Department of Life Sciences and Biotechnology, University of Ferrara, Ferrara, Italy; ^6^Internal Medicine, State Hospital, Borgo Maggiore, San Marino; ^7^School of Medicine and Surgery, University of Milano-Bicocca, Milan, Italy

**Keywords:** rheumatoid arthritis, cardiovascular risk, real-world population, cardiovascular events, prevalence

## Abstract

**Background:** Rheumatoid arthritis (RA) is associated with an increase in cardiovascular (CV) risk. This issue maybe not only explained by a genetic component, as well as by the traditional CV risk factors, but also by an underestimation and undertreatment of concomitant CV comorbidities.

**Method:** This was a retrospective matched-cohort analysis in the Italian RA real-world population based on the healthcare-administrative databases to assess the CV risk factors and incidence of CV events in comparison with the general population. Persistence and adherence to the CV therapy were also evaluated in both groups.

**Results:** In a RA cohort (*N* = 21,201), there was a greater prevalence of hypertension and diabetes with respect to the non-RA subjects (*N* = 249,156) (36.9 vs. 33.4% and 10.2 vs. 9.6%, respectively), while dyslipidemia was more frequent in the non-RA group (15.4 vs. 16.5%). Compared with a non-RA cohort, the patients with RA had a higher incidence of atrial fibrillation (incidence rate ratio, IRR 1.28), heart failure (IRR 1.53), stroke (IRR 1.19), and myocardial infarction (IRR 1.48). The patients with RA presented a significantly lower persistence rate to glucose-lowering and lipid-lowering therapies than the controls (odds ratio, *OR* 0.73 [95% *CI* 0.6–0.8] and *OR* 0.82 [0.8–0.9], respectively). The difference in the adherence to glucose-lowering therapy was significant (*OR* 0.7 [0.6–0.8]), conversely no statistically significant differences emerged regarding the adherence to lipid-lowering therapy (*OR* 0.89 [95% *CI* 0.8–1.0]) and anti-hypertensive therapy (*OR* 0.96 [95% *CI* 0.9–1.0]).

**Conclusion:** The patients with RA have a higher risk of developing CV events compared with the general population, partially explained by the excess and undertreatment of CV risk factors.

## Introduction

The pathophysiology of rheumatoid arthritis (RA) involves a complex interplay of environmental and genetic factors, leading to chronic synovial inflammation and joint damage (1). Beyond synovitis, the patients with RA are at high risk of developing cardiovascular disease (CVD) (2), as inflammation plays a pivotal role in the pathogenesis of CVD (3). Therefore, the patients with RA have approximately a double risk of atherosclerotic CVD, stroke, heart failure, and atrial fibrillation (AF) compared with the general population (2–4). Furthermore, the patients with active RA, differently from the general population, have increased CV events and mortality, paradoxically associated with the reduced circulating lipid levels (5). In fact, the lipid functions are abnormal in RA (5). Several studies have shown an increased CVD risk since the early stages of RA, and mortality linked to the CV events increases along with disease duration (6–8). As the degree of CVD involvement in RA correlates with the degree of systemic inflammation, conventional synthetic (cs) disease-modifying anti-rheumatic drugs (DMARDs) and biologic (b)DMARDs protect from the CV events (9), while chronic corticosteroids increase the CVD risk (3).

Although this high risk of CVD has been well documented and known for decades, the patients with RA still receive the suboptimal primary and secondary CVD preventive cares with respect to other high-risk subjects, and one of the most important clinical unmet needs in RA relates to improving the CVD preventive strategies (10). To note, the excess of CVD risk in the patients with RA is in part connected to an underestimation of the concomitant CV comorbidities, as well as to their suboptimal management (4), beyond the relevance of a genetic component in CVD development documented by several studies (11). In this regard, well-established risk charts, such as the Systematic COronary Risk Evaluation (SCORE) were found to underestimate the actual CV risk of patients with RA (12). Because of that, non-invasive techniques, such as the carotid ultrasound are used to identify the patients with RA who are at high risk of cardiovascular events (13).

In Italy, no solid data are available regarding the CV risk and CV risk factors in RA, as well as no data, were reported regarding the persistence and adherence to CV risk-lowering therapy. The analyses in a national context are important due to the differences in disease and patients' management. Thus, we conducted an analysis in Italian RA real-world population with the main objectives (i) to assess the CV risk factors, CV events, and overall mortality in the patients with by comparing them with the general population; (ii) to compare the persistence and adherence with the CV risk-lowering therapy in the patients with RA vs. non-RA. For these purposes, we took advantage of the RECord linkage Of Rheumatic Disease (RECORD) study promoted by the Italian Society for Rheumatology, aiming to set up a national surveillance system to monitor the health burden of rheumatic diseases in Italy.

## Patients and Methods

### Study Design

This was a retrospective matched cohort study, performed using data of the RECORD project promoted by the Italian Society for Rheumatology, aimed to implement an algorithm to identify the patients with RA using the administrative healthcare databases (AHDs) information and to measure the prevalence, incidence, and mortality of RA (14, 15). The RECORD data cover the period 2004–2013.

The data sources for the RECORD project were the AHDs of Lombardy, an Italian region with more than 10,000,000 inhabitants (about 16% of the entire Italian population). The entire Italian population is covered by the National Health Service (NHS), and in Lombardy, an automated system of AHDs has been created to collect a variety of information (16). The source registry is an electronic database that contains the fields that are built as an obligatory menu, limiting the possible errors and missing data.

The study was approved by the ethical committee of the Pavia University Hospital (deliberation of March 12, 2012) and it has been performed in accordance with the ethical standards as laid down in the 1964 Declaration of Helsinki and its later amendments.

### Study Population

The target population in the RECORD project included all the residents of Lombardy, aged 18 years and older. In the RECORD project, a cohort of patients with RA was identified applying the aforementioned algorithm (14). Each RA case was matched with the four patients with non-RA, during the same period, by gender and age. For each patient, the clinical and pharmacological history covering the period 2004–2013 was available.

In our analysis, we excluded all the subjects with a CV event that occurred between 2004 and 2008. All the patients were followed from January 2009 until the first CV event, emigration, death, or end of follow-up (31 December 2013), whichever came first.

### Variables and Statistical Analysis

The CV risk factors (hypertension, dyslipidemia, or diabetes mellitus [DM]) frequencies were extracted and the difference in terms of the proportion of subjects with CV risk factors was assessed by performing the χ^2^ test in the period between January 01, 2004, and December 31, 2008.

The CV events taken into consideration in these study analyses were myocardial infarction, ischemic stroke, heart failure, and AF, classified with the diagnosis-related group 24 (DRG-24) and the International Classification of Diseases, 9th revision-Clinical Modification (ICD9-CM) codes (Supplementary Material). If a patient presented more than one CV event during the same hospitalization, all the incident events were taken into consideration. The comparison between the incidence rates (1,000 person/years) of CV events in the patients with RA and non-RA was carried out using the univariate and multivariate Poisson models, with the following covariates included: gender, age, and CV risk factors. The subjects with hypertension, dyslipidemia, or DM as well as patients on treatment for CV risk factors were identified by means of the data relating to the therapeutic prescriptions, Anatomical Therapeutic Chemical classification system codes (ATC, Supplementary Material), or to the presence of certifications for the aforementioned diseases.

The difference between the patients in the RA group and non-RA group for CV risk factors therapies was assessed by performing the χ^2^ test. The patients not presenting a CV risk-lowering drug therapeutic discontinuity during the follow-up ≥90 days (median time frame for the renewal of prescription) were considered persistent to the drug therapy. A patient was defined as adherent to the CV risk-lowering treatment when the proportion of day covered (PDC) by the treatment for at least 80% of his/her follow-up, taking into account multiple treatments for a specific indication (17). In case of treatment overlap, the overlapping days were considered once. To calculate the PDC, the amount of drug purchased by the patient was considered and this quantity was divided by the defined daily dose.

Three logistic regression models were applied, defining persistence or adherence to the therapy as outcome and presence or absence of RA as covariate adjusting for age and sex. The association estimates were reported as odds ratio (*OR*) and relative 95% *CI*. No formal sample size estimation was made for this observational retrospective study as we analyzed all the eligible RA subjects with a high number of matched non-RA cases.

All the hypothesis tests were two-sided and the *p*-values for statistical significance were set at 0.05. All the analyses were performed using an R statistical software 3.3 version (Foundation for Statistical Computing, Vienna, Austria).

## Results

### The CV Events and CV Risk Factors

The RA cohort consisted of 21,201 patients with 16,098 (76%) women and a median age of 61.7 years (±13.7), whereas the non-RA cohort was represented by 249,156 subjects with 179,407 (72%) women and a median age of 62.5 years (±14.4). Regarding the presence of CV risk factors, rates of hypertension and DM were higher in the patients with RA (36.9 vs. 33.4%, *p* < 0.001, and 10.2 vs. 9.6%, *p* = 0.004, respectively), while the rate of dyslipidemia was higher in the patients with non-RA (16.5 vs. 15.4%, *p* < 0.001).

Among the patients with RA, 1,769/21,201 (8.3%) subjects had at least one CV event during the follow-up, whereas 16,381/249,156 (6.6%) non-RA subjects had at least one CV event in the same period.

Some subjects had simultaneously (i.e., in the same hospitalization) more than one CV event. Among the RA cases, 1,618 subjects had one event and 151 had two events. Among the non-RA cases, 14,661 subjects had only one event, 1,707 had two events, and 13 had three events.

The development of the first CV event was associated with the presence of each CV risk factor and RA acted as an independent risk factor for the development of the first CV event (incidence rate ratio [IRR] 1.39 [1.3–1.5], *p* < 0.001). In addition, the patients with RA have an increased risk of death compared with the non-RA subjects from any cause (IRR 1.35 [1.3–1.4] *p* < 0.001). Two thousand and nineteen patients with RA (9.5%) and 20,273 (8.1%) non-RA subjects deceased during the follow-up.

More specifically, the incidence rates of AF, heart failure, and acute myocardial infarction were significantly higher in the patients with RA than in the non-RA group (IRR 1.17, 1.35 and 1.39, respectively; *p* < 0.001) (Table 1). In addition, a higher incidence of stroke occurred in the RA group, with a *p*-value not statistically significant (crude IRR: 1.10 [*CI* 1.0–1.3], *p* = 0.171).

**Table 1:** Incidence rates of cardiovascular (CV) events in the patients with and without RA (per year, per 1,000 person).

**CV event**	**RA, N**	**RA, IR [95%CI]**	**non-RA, N**	**non-RA, IR [95%CI]**	**IRR**	***p*-value**
Atrial fibrillation	687	7.01 [5.9–8.1]	6,964	5.98 [5.7–6.3]	1.17 [1.1–1.3]	<0.001(^***^)
Heart failure	402	4.10 [3.2–5.0]	3,546	3.04 [2.8–3.3]	1.35 [1.2–1.5]	<0.001(^***^)
Myocardial infarction	603	6.15 [5.1–7.2]	5,140	4.41 [4.2–4.7]	1.39 [1.3–1.5]	<0.001(^***^)
Stroke	228	2.33 [1.7–3.0]	2,464	2.11 [1.9–2.3]	1.10 [1.0–1.3]	0.171

*CV, cardiovascular; CI, confidence interval; IR, incidence rate; IRR, incident rate ratio; RA, rheumatoid arthritis; IRR, were adjusted for age, sex, and CV risk factors*.

*** *Highly significant*.

The presence of CV comorbidities, such as hypertension and DM lead to an increase in the adjusted incidence of AF (IRR 2.03 and 1.18, respectively; all *p* < 0.001), whereas the subjects with dyslipidemia had a risk of developing AF similar to those not affected by this comorbidity (0.99, *p* = 0.663).

The patients with RA, DM, or hypertension had a significantly higher risk of developing heart failure (IRR 1.54, 1.82, and 1.62, respectively; *p* < 0.001), while dyslipidemia did not act as an independent CV risk factor for the development of this event (IRR 1.02, *p* = 0.665).

The presence of RA, DM, hypertension, or dyslipidemia correlated independently with the risk of developing myocardial infarction (IRR 1.48, 1.50, 1.57, and 1.58, respectively; *p* < 0.001).

Regarding stroke, the patients with RA had an increased risk of developing this CV event compared with the control group when adjusting for pre-specified confounders (IRR 1.19, *p* = 0.012).

All details matching the CV events and CV risk factors for both groups are reported in Table 2.

**Table 2:** Assessment of the relationship between CV risk factors and CV events.

	**Atrial fibrillation**	**Heart failure**	**Stroke**	**Myocardial infarction**
	**IRR [95% CI]**	**IRR [95% CI]**	**IRR [95% CI]**	**IRR [95% CI]**
RA	1.28 [1.2–1.4]	1.54 [1.4–1.7]	1.19 [1.0–1.4]	1.48 [1.4–1.6]
Hypertension	2.03 [1.9–2.1]	1.82 [1.7–2.0]	1.49 [1.4–1.6]	1.57 [1.5–1.6]
DM	1.18 [1.1–1.3]	1.62 [1.5–1.7]	1.52 [1.4–1.7]	1.50 [1.4–1.6]
Dyslipidemia	0.99 [0.9–1.0]	1.02 [0.9–1.1]	0.98 [0.9–1.1]	1.58 [1.5–1.7]

*DM, diabetes mellitus; IRR, incidence rate ratio; RA, rheumatoid arthritis*.

### Persistence and Adherence

Regarding treatment for the CV risk factors, 80% patients with RA and 80.2% patients with non-RA with hypertension had at least one prescription of anti-hypertensive drug (*p* = 0.677), among the patients with DM, 73.3% RA vs. 80.9% non-RA had hypoglycemic treatment (*p* < 0.001), and among the patients with dyslipidemia, 71.8% RA vs. 77.2% non-RA (*p* < 0.001) had at least one prescription for lipid-lowering therapies. No statistically significant differences occurred in the two groups regarding the therapeutic persistence for antihypertensive drugs (44 vs. 45%, *OR* 1.01 [0.9–1.1], *p* = 0.78). On the other hand, the patients with DM-RA had a lower persistence to hypoglycemic treatment (46 vs. 54%, *OR* 0.73 [0.7–0.8], *p* < 0.001), and dyslipidemia RA to lipid-lowering therapies (29 vs. 33%, 0.82 [0.8–0.9], *p* < 0.001) than the non-RA group.

No statistically significant differences emerged regarding the adherence to lipid-lowering therapy (*OR* 0.89 [0.8–1.0], *p* = 0.054) and antihypertensive therapy (*OR* 0.96 [0.9–1.0], *p* = 0.179) between the two groups, whereas the patients with RA showed less adherence to the glucose-lowering treatments than the non-RA subjects (*OR* 0.70 [0.6–0.8], *p* < 0.001).

## Discussion

The patients with RA have increased mortality and morbidity compared with the general population (18). There are no univocal reports that explain these data, as they vary according to the type of cohort and the care setting examined. Nevertheless, the increase in mortality in RA is largely attributable to the CV events (19).

In Italy, solid data are not available regarding the distribution of CV risk factors and their correlation with the onset of new CV events in patients with RA. The present analysis aimed to fill this gap and to investigate if the CV events are a consequence of suboptimal drug utilization.

Regarding the presence of CV risk factors, in the analyzed cohort there was a greater prevalence of hypertensive subjects in the patients with RA (36.9 vs. 33.4%). A wide variability regarding the prevalence of hypertension in the patients with RA has been documented (20, 21): a higher prevalence of hypertension among the patients with RA could be affected by the lack of analysis of confounding variables because of the comparison of patients coming from different care settings (20). For example, the prevalence of hypertension and diabetes was found to be increased in the Spanish individuals with RA compared with matched controls (22). Glucocorticoid-induced DM might partially explain the excess diagnosis of DM in the patients with RA.

The prevalence of dyslipidemia in the RA group (15.4 vs. 16.5%) was significantly lower than in the control group and this data could be explained with the “lipid paradox” (23, 24), however, it was not possible to assess the disease activity in the population under examination. Our RA cases have a higher prevalence of DM than the control group (10.2 vs. 9.6%). From the analysis of the data available in the literature, the conflicting elements emerged: a 2011 meta-analysis documented an increased prevalence of DM in the patients with RA (25), whereas other studies did not show statistically significant differences in the prevalence of DM in women with or without RA (26). According to our data, the baseline data from the Spanish inflammatory arthritis registry Cardiovascular in rheumatology (CARMA) showed a lower prevalence of hypercholesterolemia than the matched controls (22).

During the follow-up, 18,150 patients presented at least one CV event: 1,769 (8.3%) among the RA group and 16,381 (6.6%) in the non-RA group, respectively. Compared with the control group, the patients with RA have a 30% higher overall incidence of CV events. These data appear superimposable to those available in the literature that the patients with RA have also a higher incidence of AF, heart failure and myocardial infarction, and stroke compared with the control group, in line with data previously reported in the patients with RA (27–30). To note, data after 5 years of follow-up from the prospective CARMA project in patients with inflammatory arthritis, unlike expected, showed that the frequency of CV events did not increase in the patients with RA compared with the control groups. At that time, the CARMA project member speculated on the protective effect of the biologic therapy administered to a high number of patients, due to the favorable effect of biologics on the insulin resistance, lipid composition, and other beneficial metabolic effects mediated by these agents, as well as the effect on reducing inflammation. Likewise, the greater knowledge of the EULAR recommendation for the management of traditional CV risk factors among the members of the CARMA project may also have explained these favorable results (31). The discrepancy supports the gap in proper stratification and treatment of the patients with RA in our cohort.

From the results of our study, RA, hypertension, and DM act as the independent risk factors for the development of AF and heart failure. Furthermore, the presence of RA acts as an independent risk factor for the development of myocardial infarction and for mortality, indicating that disease-specific risk factors play an important role.

Evaluating the data regarding pharmacoutilization of drugs for CVD, it emerged that the patients with RA have lower persistence to the glucose-lowering and lipid-lowering treatments than the control group, and, when persistent, are less adherent to glucose-lowering therapy, confirming the previous reports (32). These results indicate that careful monitoring for DM and hyperlipidemia for persistent treatments of related drugs are needed in the patients with RA.

The main strengths of the study are represented by the absence of selection bias, the large sample size with no loss to follow-up, and the duration of the follow-up. Furthermore, this report presents the largest matched-cohort study on CV risk in the patients with RA and non-RA ever reported in Italy with no missing information as the RECORD database covers all the events of interest generated by the target population. The main limitation is linked by the retrospective design of an administrative database. In fact, the use of data extracted from the administrative databases lacks some clinical information that could influence the CV risk. An essential part of CV risk management consists of a screening of five traditional parameters: blood pressure, smoking status, body weight, blood glucose, and lipid profile (33, 34). For the present analyses, data on smoking status and body weight were not available. In addition, the acquired data are limited to the actually delivered prescriptions and no information was available regarding the reasons for primary non-adherence to the treatments or for the suspension of the therapies. To note that we analyzed data registered only during hospitalizations with underestimation of events due to the lack of events that occurred both in the out-patient regimen and in patients deceased before the hypothetical hospitalization.

The differences in treatment and CV risk factors only partially justify the increase in CVD risk that could be explained by the pathophysiological characteristics of RA.

Considering the results of our study that ascertain a central role of RA and the traditional CV risk factors in the onset of new CV events, it is essential to translate into practice the programs of surveillance and management of traditional CV risk factors, proper CV risk assessment taking into account the disease-specific characteristics (i.e., lipid paradox) as well as in assessing the adherence of patients to CVD risk-lowering therapy.

In conclusion, data emerging from this study confirm that the patients with RA have a higher risk of developing CV events including AF, heart failure, stroke, and myocardial infarction, as well as an increased mortality rate compared with the general population, even independently from the presence of other CV comorbidities. CV risk factor management should be an essential part of the care of patients with RA. Although relevant international guidelines exist, there are still major gaps in the knowledge and risk factor management implementation in these patients' groups and CV risk should be assessed according to the national guidelines as they may differ among the countries.

## Data Availability Statement

The raw data supporting the conclusions of this article will be made available by the authors, without undue reservation.

## Ethics Statement

The studies involving human participants were reviewed and approved by the Ethical Committee of the Pavia University Hospital (deliberation of 12/March/2012) and it has been performed in accordance with the ethical standards as laid down in the 1964 Declaration of Helsinki and its later amendments. The patients/participants provided their written informed consent to participate in this study.

## Author Contributions

AZan, CS, AZam, and GC: study conception and design. AZan, CS, GG, ES, and GC: acquisition of data. LA, AZan, CS, AZam, and GC: analysis and interpretation of data. CS had full access to all the data in the study and takes responsibility for the integrity of the data and the accuracy of the data analysis. All authors were involved in drafting the article or revising it critically for important intellectual content, and approved the final version to be submitted for publication.

## Funding

The study was supported by the Italian Society for Rheumatology.

## Conflict of Interest

The authors declare that the research was conducted in the absence of any commercial or financial relationships that could be construed as a potential conflict of interest.

## Publisher's Note

All claims expressed in this article are solely those of the authors and do not necessarily represent those of their affiliated organizations, or those of the publisher, the editors and the reviewers. Any product that may be evaluated in this article, or claim that may be made by its manufacturer, is not guaranteed or endorsed by the publisher.
