# Supplementary material for: Rheumatoid Arthritis and Cardiovascular Risk: Retrospective Matched-Cohort Analysis Based on the RECORD Study of the Italian Society for Rheumatology
Source: Front Med (Lausanne). 2021 Oct 5;8:745601. doi: 10.3389/fmed.2021.745601 (PMC8523847; doi:10.3389/fmed.2021.745601)
Supplement: Supplementary file 1 [file Data_Sheet_1.docx]

Supplementary Material

# Diagnosis-related group 24 (DRG-24) and International Classification of Diseases, 9th revision - Clinical Modification (ICD9-CM) codes for cardiovascular events

# Myocardial infarction

# DRG-24 code: 121, 122, 123, 140

# ICD9-CM: 410*, 411*,413*

# Stroke

# ICD9-CM: 434*

# Heart failure

# DRG-24 code: 127

# ICD9-CM: 4289

# Atrial fibrillation

# DRG-24 code: 139

# ICD9-CM: 42731

# Diagnosis certification and Anatomical Therapeutic Chemical (ATC) classification system codes for cardiovascular risk factors

# Hypertension

# Diagnosis certification: 031

# ATC code: C09AA01 ,C09AA02 ,C08DA01, C09CA01, C09CA03, C09CA04, C08CA01, C08CA05, C08CA13, C08CA07, C09AA04,C09AA03,C03BA11, C03AA03, C03DB01, C07AB02, C07AB03, C07AB07, C07AB12, C07AG02

# Diabetes Mellitus

# diagnosis certification: 013

# ATC code: A10B*, A10A*

# Dyslipidemia

# diagnosis certification 025

# ATC code: C10AA*
